# Cingulate dynamics track depression recovery with deep brain stimulation

**DOI:** 10.1038/s41586-023-06541-3

**Published:** 2023-09-20

**Authors:** Sankaraleengam Alagapan, Ki Sueng Choi, Stephen Heisig, Patricio Riva-Posse, Andrea Crowell, Vineet Tiruvadi, Mosadoluwa Obatusin, Ashan Veerakumar, Allison C. Waters, Robert E. Gross, Sinead Quinn, Lydia Denison, Matthew O’Shaughnessy, Marissa Connor, Gregory Canal, Jungho Cha, Rachel Hershenberg, Tanya Nauvel, Faical Isbaine, Muhammad Furqan Afzal, Martijn Figee, Brian H. Kopell, Robert Butera, Helen S. Mayberg, Christopher J. Rozell

**Affiliations:** 1 School of Electrical and Computer Engineering, Georgia Institute of Technology, Atlanta, GA USA; 2 Nash Family Center for Advanced Circuit Therapeutics, Icahn School of Medicine at Mount Sinai, New York, NY USA; 3 Department of Radiology, Icahn School of Medicine at Mount Sinai, New York, NY USA; 4 Department of Neurosurgery, Icahn School of Medicine at Mount Sinai, New York, NY USA; 5 Department of Psychiatry and Behavioral Sciences, Emory University School of Medicine, Atlanta, GA USA; 6 Wallace H. Coulter Department of Biomedical Engineering at Georgia Institute of Technology and Emory University, Atlanta, GA USA; 7 Emory University School of Medicine, Atlanta, GA USA; 8 Department of Psychiatry, Schulich School of Medicine and Dentistry at Western University, London, Ontario Canada; 9 Department of Neuroscience, Icahn School of Medicine at Mount Sinai, New York, NY USA; 10 Department of Psychiatry, Icahn School of Medicine at Mount Sinai, New York, NY USA; 11 Department of Neurosurgery, Emory University School of Medicine, Atlanta, GA USA; 12 Department of Neurology, Emory University School of Medicine, Atlanta, GA USA; 13 Department of Neurology, Icahn School of Medicine at Mount Sinai, New York, NY USA

**Keywords:** Depression, Depression, Translational research, Prognostic markers

## Abstract

Deep brain stimulation (DBS) of the subcallosal cingulate (SCC) can provide long-term symptom relief for treatment-resistant depression (TRD)^1^. However, achieving stable recovery is unpredictable^2^, typically requiring trial-and-error stimulation adjustments due to individual recovery trajectories and subjective symptom reporting^3^. We currently lack objective brain-based biomarkers to guide clinical decisions by distinguishing natural transient mood fluctuations from situations requiring intervention. To address this gap, we used a new device enabling electrophysiology recording to deliver SCC DBS to ten TRD participants (ClinicalTrials.gov identifier NCT01984710). At the study endpoint of 24 weeks, 90% of participants demonstrated robust clinical response, and 70% achieved remission. Using SCC local field potentials available from six participants, we deployed an explainable artificial intelligence approach to identify SCC local field potential changes indicating the patient’s current clinical state. This biomarker is distinct from transient stimulation effects, sensitive to therapeutic adjustments and accurate at capturing individual recovery states. Variable recovery trajectories are predicted by the degree of preoperative damage to the structural integrity and functional connectivity within the targeted white matter treatment network, and are matched by objective facial expression changes detected using data-driven video analysis. Our results demonstrate the utility of objective biomarkers in the management of personalized SCC DBS and provide new insight into the relationship between multifaceted (functional, anatomical and behavioural) features of TRD pathology, motivating further research into causes of variability in depression treatment.

## Abstract (web summary)

This study demonstrates how activity in the cingulate cortex tracks depression recovery, providing symptom relief using deep brain stimulation.

## Main

Patients with treatment-resistant depression (TRD) experience a wide variety of debilitating symptoms, including persistent negative mood, anhedonia, psychomotor retardation and suicidal thoughts. While many patients with TRD who receive experimental subcallosal cingulate (SCC) deep brain stimulation (DBS) have responded to continuous stimulation with durable symptom relief^4–8^, the clinical management of these patients is often complex due to a number of interacting factors. In particular, the progress of antidepressant response is nonlinear and different for each individual^1,2^, often involving periods of mood fluctuation for which there is no absolute unanimous clinical interpretation. Without objective markers of depression severity, psychiatrists rely on clinical intuition to decide whether to change stimulation parameters or apply a watchful waiting approach. Currently, clinical teams use interviews and symptom surveys such as the Hamilton Depression Rating Scale (HDRS) to quantify depression severity, but these gold-standard rater-dependent measures are often obscured by various non-specific factors such as subjective recall biases^9,10^ and reactions to environmental circumstances. For example, while depression diagnostic criteria are based on negative mood and anhedonia that are sustained over a period of weeks, patients may experience normal transient short-term mood fluctuations due to a variety of factors (for example, stressful life events, interrupted sleep or transitory anxiety) that are reflected in the HDRS and confound the measurement of core depression symptom changes. Therefore, objective markers of brain changes underlying DBS-mediated recovery are necessary to standardize treatment approaches and aid in scaling SCC DBS to an approved therapy for TRD.

**Fig. 1:**
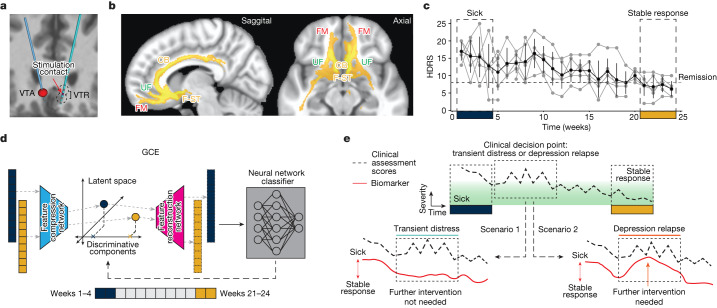
Overview of study procedures. **a**, Coronal view of the DBS lead targeting bilateral SCC in an example patient. The red sphere indicates the volume of tissue activated (VTA) with the final stimulation parameters. The black circles indicate the volume of tissue recorded (VTR) from each electrode contact, showing coverage of grey matter that are the likely sources of the recorded LFP. **b**, Common activation pathway patterns from chronic stimulation VTA seed of the six participants at 6 months. **c**, Trajectory of HDRS-17 scores over 24 weeks for five participants (of six total) who were typical responders. Grey lines indicate individuals and the black line indicates the mean. Error bars indicate standard deviation (*n* = 5 participants). Clinical consensus was that all five were ‘sick’ during weeks 1–4 and in ‘stable response’ during weeks 20–24. **d**, Schematic of deriving the SDC from LFP features. A neural network classifier is first trained with data from the ‘sick’ and ‘stable response’ states of all typical responders. Next, separate neural networks are trained to compress the data from the spectral feature space to a low-dimensional latent space and then reconstruct the data from that compressed version. Using recent advances in XAI techniques, one of these latent dimensions is a discriminative component constrained to represent the simultaneous data changes (the SDC) used by the classifier to distinguish ‘sick’ from ‘stable response’. **e**, Illustration of the utility of an objective biomarker. When patients experience instability in symptom scores, decisions about treatment (for example, stimulation voltage adjustment) must be made by inferring whether the instability is due to transient distress (scenario 1) or depression relapse (scenario 2). A biomarker that reflects progress of the brain towards ‘stable response’ will enable more effective clinical decision-making about interventions. CB, cingulum bundle; UF, uncinate fasciculus; FM, forceps minor; F-ST, frontostriatal fibres.

We address this gap by leveraging several simultaneous advances to derive a data-driven brain-based biomarker of stable depression recovery that can be used to differentiate clinically acute scenarios from periods of normal transient distress (illustrated in Fig. 1e). First, the high response rate combined with the heterogeneous trajectories to recovery achieved with this clinical cohort provides a unique opportunity to explore intersubject differences in the path to achieving antidepressant response. Second, while most of the current understanding of SCC electrophysiology dynamics in DBS arises from acute measurements (for example, intraoperative/perioperative local field potentials (LFPs)), new neurotechnology platforms allowing long-term electrophysiology monitoring provided an unprecedented opportunity to study longitudinal changes with DBS over a 24-week treatment period as patients achieved stable recovery. Third, recent explainable artificial intelligence (XAI) developments have introduced approaches to explaining ‘black box’ machine learning models, providing a powerful framework for the data-driven discovery of effective biomarkers.

This Article demonstrates a biomarker that accurately identifies depressive and recovered states, tracks individual recovery trajectories and predicts relapses, provides evidence of differential acute and sustained neuronal network adaptations and is concordant with objective changes in facial expression over the course of recovery. Furthermore, a multimodal analysis based on this brain signal shows that specific structural and functional deficits in the targeted white matter network reflect baseline disease severity (number of lifetime depressive episodes) and time to respond to DBS, demonstrating individual differences that account for variable recovery trajectories with SCC DBS. Taken together, these results advance the existing practice of SCC DBS by providing actionable objective information to support personalized clinical management, provide new insight into the complex relationship between functional, structural and behavioural factors involved in patient-specific recovery, and motivate further research in using multimodal measurements to advance the treatment of depressive disorders.

## Clinical outcomes of cohort

The study cohort consisted of ten consecutively recruited participants who were implanted with an experimental DBS implanted pulse generator (IPG) that served both stimulation and recording functions. DBS leads were inserted at the intersection of four major white matter pathways (Fig. 1a,b) identified from earlier studies^11,12^. All participants met study inclusion criteria before implantation with a minimum depression severity HDRS-17 score equal to or higher than 20 (Extended Data Table 1). Stimulation was turned on following a 4-week postsurgery recovery phase, and the primary endpoint of the study was defined as the HDRS-17 score at 24 weeks of chronic SCC DBS. At a cohort level, participants experienced a significant reduction in HDRS-17 score from the presurgery baseline with a mean HDRS-17 of 22.3 (s.d. 1.64), to the end of the 24-week observation phase with a mean HDRS-17 of 7.3 (s.d. 3.62). At an individual level, nine out of ten participants were deemed to be responders (greater than 50% decrease in HDRS-17) and 7 out of 10 were deemed to be in remission (HDRS-17 less than 8). Despite the consistent clinical outcomes at the 24-week endpoint, individual patients showed variable recovery trajectories, with some achieving clinical response much earlier than others (Fig. 1c).

**Extended Data Fig. 1:**
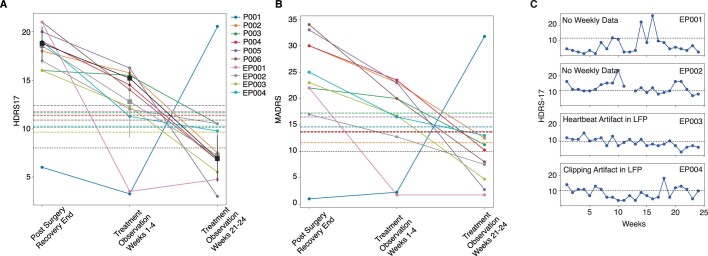
Clinical assessment scores across different phases. **a**, HDRS-17 scores across different phases. Dashed lines indicate the score at which the participant is considered to be a responder (based on 50% decrease in HDRS-17). Dotted line indicates a HDRS-17 score of 8 below which participants are considered to be in remission. **b**, MADRS scores across different phases. Dashed lines indicate the score at which the participant is considered to be a responder (based on 50% decrease in MADRS). Dotted line indicates a MADRS score of 10 below which participants are considered to be in remission. **c**, HDRS-17 Trajectories of participants excluded from analysis.

Chronic electrophysiological data for analyses were available for six of the ten participants. Of these participants, five of the six demonstrated a typical response trajectory (‘typical responders’). The five participants entered the 24-week observation phase with a mean HDRS-17 of 18.80 (s.d. 1.72) reflecting a mean decrease of 4.4 (s.d. 2.15) following surgery and intraoperative stimulation. After 4 weeks of chronic stimulation, these ‘typical responders’ experienced a further decrease with a mean HDRS-17 of 15.20 (s.d. 0.83) (mean decrease 3.6, paired one-sided Wilcoxon signed-rank test, *P* = 0.031), and in weeks 21 to 24 their mean HDRS-17 was 6.92 (s.d. 2.39) (Extended Data Fig. 1a). The difference in HDRS-17 between the first 4 weeks and the last 4 weeks was statistically significant (mean decrease 8.3, paired one-sided Wilcoxon signed-rank test, *P* = 0.031). At the end of 24 weeks, these five participants reached clinical responder status, and four out of the five participants achieved remission. Based on the weekly HDRS-17 scores, all participants were considered to be in a ‘sick’ state during the first 4 weeks and in a ‘stable response’ state during the last 4 weeks of this period. A similar trend was observed in scores from another standard depression rating scale, the Montgomery-Åsberg Depression Rating Scale (MADRS, Extended Data Fig. 1b). The recovery trajectories of participants whose electrophysiological data were not available were qualitatively similar to those of participants included in this study (Extended Data Fig. 1c).

## SCC dynamics delineate the depression state

**Fig. 2:**
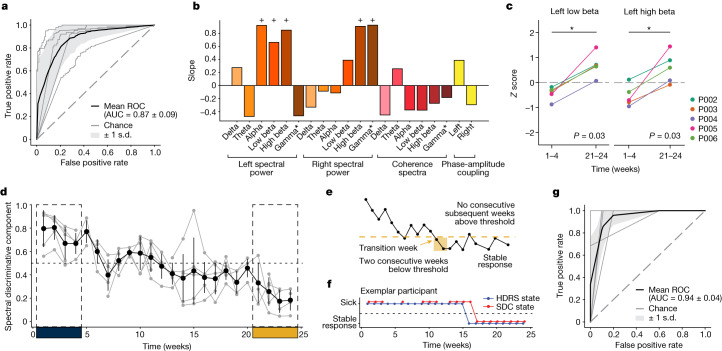
Identification and performance of SDC. **a**, ROC curves of the LFP classifier in classifying ‘sick’ and ‘stable response’ states with leave-one-participant-out cross-validation. Grey lines indicate the ROC curve of individual folds of the cross-validation. Black line indicates the mean ROC curve. **b**, Simultaneous change in spectral features that capture the difference between the ‘sick’ and ‘stable response’ states as reflected by the SDC. The + symbol indicates the top five discriminative features. Gamma* indicates the 30–40 Hz band, as described in the text. **c**, Change in left-low beta and left-high beta power from the beginning to the end of the observation phase (relative to last week of postsurgical period without stimulation). *P = 0.031 (one-sided Wilcoxon signed-rank test). **d**, Trajectory of the SDC over 24 weeks. Grey lines indicate individual participants and the black line indicates the average of the five typical responders. Error bars indicate standard deviation (*n* = 5 participants). **e**, Illustration of identifying state change from ‘sick’ to ‘stable response’. Transition to ‘stable response’ is defined as the week when the measure falls below the transition threshold for two consecutive weeks and (during the observation period) never returns above threshold for two or more weeks. **f**, State change from ‘sick’ to ‘stable response’ in an exemplar participant (P002). The blue line indicates the state inferred from HDRS-17 scores and the red line indicates the state inferred from the SDC. **g**, ROC curves of the SDC state predicting the HDRS state. Grey lines indicate the ROC curve for individual participants and the black line indicates the mean ROC curve.

**Extended Data Fig. 2:**
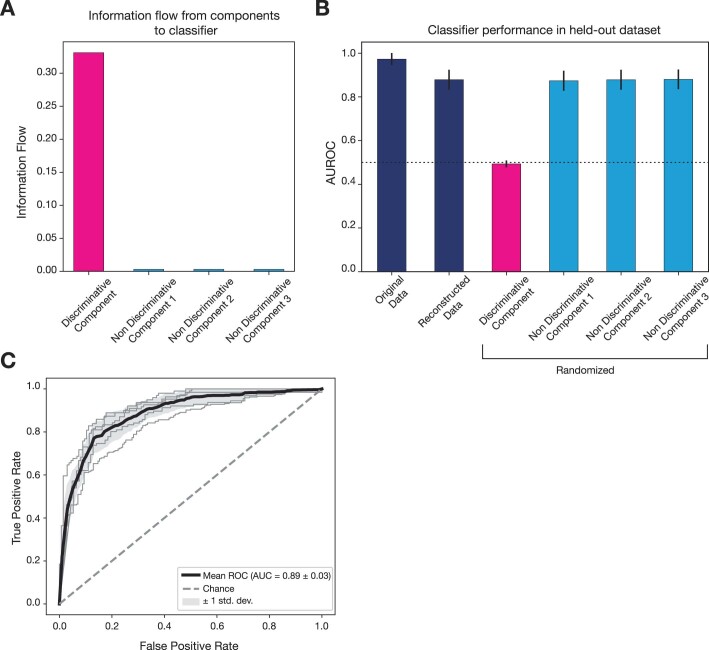
Validation of Generative Causal Explainer (GCE). **a**, Information flow from low-dimensional latent space components to classifier prediction indicates that classifier prediction is affected by the discriminative component and not by the non discriminative components. **b**, Classifier performance in leave-one-participant-out cross-validation for different datasets. Reconstructed data refers to data reconstructed from GCE using all components. Performance of the classifier in datasets reconstructed by randomizing discriminative and non-discriminative components is shown in magenta and cyan bars. Randomizing the discriminative component of the held-out dataset affected the classifier performance significantly, indicating that the association between data and classifier prediction is impaired, which in turn confirmed that the GCE did not overfit to the training dataset. Grey stars denote AUROC for each fold of the cross-validation. Error bars indicate standard deviation (*n* = 5 cross-validation fold). **c**, Receiver operating characteristic curve for neural network classifier trained on the reconstructed data to distinguish ‘sick’ versus ‘stable response’ state.

We extracted spectral features from LFP recorded with stimulation turned off for the classification of ‘sick’ versus ‘stable response’ (that is, the first 4 weeks and the last 4 weeks of the 24-week observation period) in the typical responders. A neural network classifier (with leave-one-participant-out cross-validation) was able to distinguish the ‘sick’ and ‘stable response’ states (area under the receiver operating characteristic (ROC; AUROC): 0.87 ± 0.09; Fig. 2a) in the five typical responders, suggesting recovery from depression is reflected in similar electrophysiological changes across participants. The parameters of the neural network classifier are provided in Extended Data Table 2. We then trained a generative causal explainer (GCE)^13^ to identify the spectral discriminative component (SDC), which is a low-dimensional latent representation of the spectral features that collectively capture the difference between the ‘sick’ and ‘stable response’ states as determined by the neural network classifier. Thus, the SDC serves as an LFP marker reflecting the status relative to binary depressive/recovered states, with higher values indicating the ‘sick’ state and lower values indicating the ‘stable response’ state. The procedure for validating the GCE model is detailed in Methods and Extended Data Fig. 2. In addition, details of the training and testing data used for training the classifier and GCE models are provided in Extended Data Table 3.

## Beta band is differentially modulated

**Extended Data Fig. 3:**
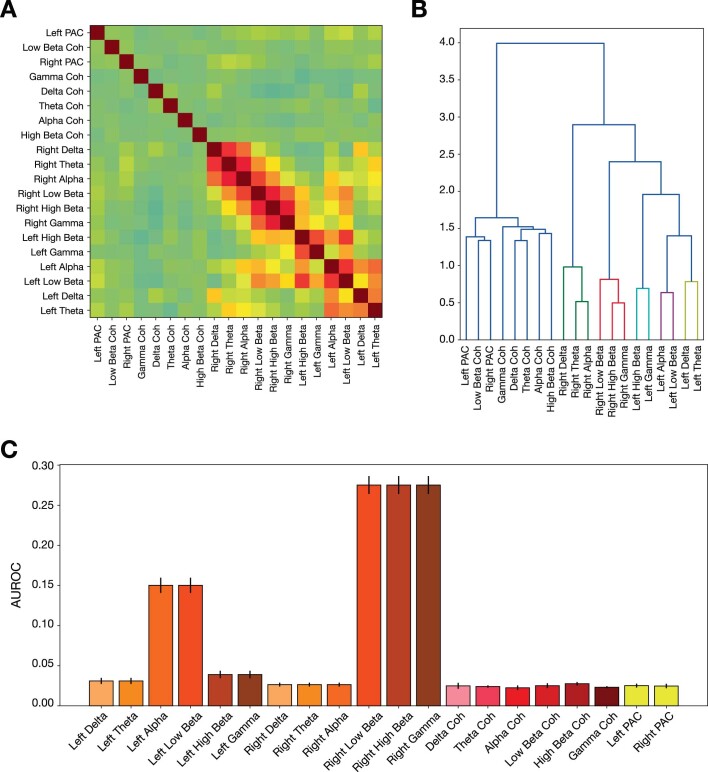
Permutation feature importance. Permutation feature importance is a shuffle-based technique to determine the contribution of features to classification performance^67^. Since the features were correlated, a dendrogram-based clustering was used to identify clusters of features (distance threshold = 1). Features within a cluster were permuted jointly to generate shuffled datasets (*n* = 100) which were then evaluated using the classifier trained on the original dataset. The decrease in performance of the shuffled datasets provides a measure of the feature’s contribution to classifier performance. **a**, Adjacency matrix based on Spearman correlation between spectral features. Hotter colors indicate a positive correlation. **b**, Dendrogram-based clustering of features. **c**, Difference in Area under ROC curve between classifier trained on original dataset and shuffled datasets (*n* = 100).

We used the slope of the joint changes in LFP features when the SDC was varied to identify the concurrent spectral features that exhibited the most changes when patients transitioned from ‘sick’ to ‘stable response’ (Fig. 2b). A positive slope indicates an increase in the feature’s magnitude when the SDC changes from the ‘sick’ state to the ‘stable response’ state, while a negative slope indicates a decrease in the feature’s magnitude. Changes in the SDC resulted in changes in many spectral features simultaneously, with the largest changes observed in left alpha (8−13 Hz), left-low beta (13−20 Hz), left-high beta (20−30 Hz), right-high beta and right-gamma band power (30−40 Hz). All these features exhibited an increase, suggesting the difference between ‘sick’ and ‘stable response’ states is driven by a bilateral increase in beta/gamma power in SCC. As a secondary confirmation, a similar subset of features was identified to be important for classification using a clustering-based permutation feature importance method (Extended Data Fig. 3).

**Extended Data Fig. 4:**
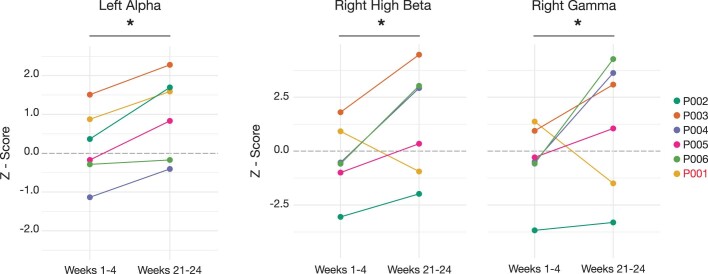
Difference between early and late changes in other relevant features. Change in power relative to the last week of post-surgical period without stimulation from the beginning of the observation phase to the end of the observation phase.

While the identified features (especially beta band power) have been previously reported to respond to stimulation in acute stimulation experiments, the current longitudinal analysis reports the opposite change pattern. Specifically, acute intraoperative SCC stimulation has been shown to decrease beta band power poststimulation offset^14,15^, whereas chronic stimulation here promotes sustained increases in beta band power. To directly compare findings here with these previous studies, we computed the beta band power across the 24-week observation phase relative to the last week of the 4-week postsurgery recovery phase (when stimulation remained off). Relative to the postsurgery off baseline, left-low beta band power (13–20 Hz) was lower in the early phase of active treatment (week 1–4 stimulation on) (one-sided Wilcoxon signed-rank test, *P* = 0.031) and higher in the late phase (week 21–24 stimulation on) (one-sided Wilcoxon signed-rank test, *P* = 0.031) in all five typical responders (Fig. 2c). The difference between the early changes and the late changes was also statistically significant (paired one-sided Wilcoxon signed-rank test, *P* = 0.031). A similar difference between the early and late changes was observed in left-high beta band power (*P* = 0.031), although the early treatment decrease and late treatment increase were not statistically significant (*P* = 0.062). This indicates that while the early effect of stimulation is consistent with the acute effects observed in previous studies, the long-term effect is distinct and in the opposite direction. While other bands with significant longitudinal changes (captured by the SDC) exhibit an increase from weeks 1–4 to weeks 21–24, only the low beta band activity exhibits the differential response of acute decrease followed by an increase with chronic stimulation (Extended Data Fig. 4).

## SDC tracks progress to stable response

**Extended Data Fig. 5:**
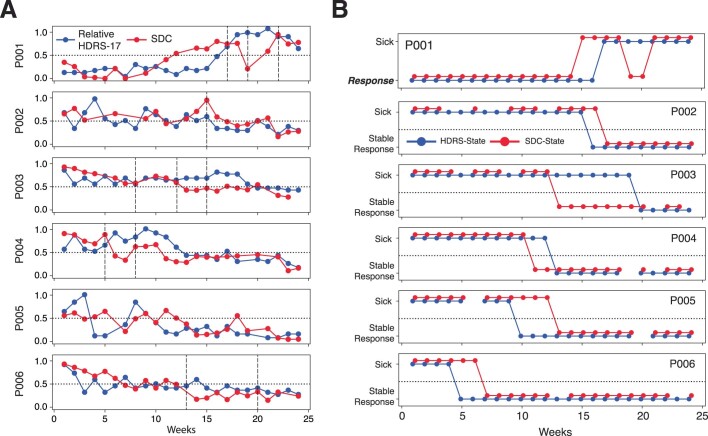
Trajectories of HDRS-17 and the SDC. **a**, Trajectory of relative HDRS-17 and spectral discriminative component for individual participants. P001 is the relapsed responder. The vertical dashed line indicates the week when the stimulation voltage was increased. **b**, Illustration of how well the SDC sick/stable response designation matches the HDRS-defined sick/stable response state in typical responders. The blue line denotes the HDRS state while the red line denotes the SDC state using a threshold of 0.5.

We computed the SDC for the intermediate period (weeks 5−20) to estimate the trajectory of LFP changes from the ‘sick’ state to the ‘stable response’ state in all patients (Fig. 2d; Extended Data Fig. 5a). To verify whether the SDC indeed tracked depressive symptoms, we compared the depressive state estimated from the SDC against the state derived from HDRS-17. We further define ‘stable response’ as the occurrence of two or more consecutive weeks of therapeutic response, followed by the absence of a subsequent loss of response (Fig. 2e). The time of stable response is taken (in retrospective analysis) to be the first week a patient reached this ‘stable response’ state. Thus, the participants are considered to be in the ‘sick’ state in the weeks preceding this time point and in the ‘stable response’ state in the weeks following this time point. Using analogous criteria on the SDC, we examined the ability of the electrophysiology marker to detect this sick/stable response state on a weekly basis, as shown in (Fig. 2f) with a ROC. When evaluated using the area under the curve (AUC) for each participant (Fig. 2g), we found this approach yields high accuracy; in weekly estimates, the SDC state matched the HDRS state over 90% of the time (AUC 0.94 ± 0.036), indicating that SDC significantly and reliably captures clinically meaningful depression states of the participants. Extended Data Figure 5b shows how well the state derived from SDC using a threshold of 0.5 tracks the state derived from HDRS-17.

## Stimulation voltage changes alter SDC

**Fig. 3:**
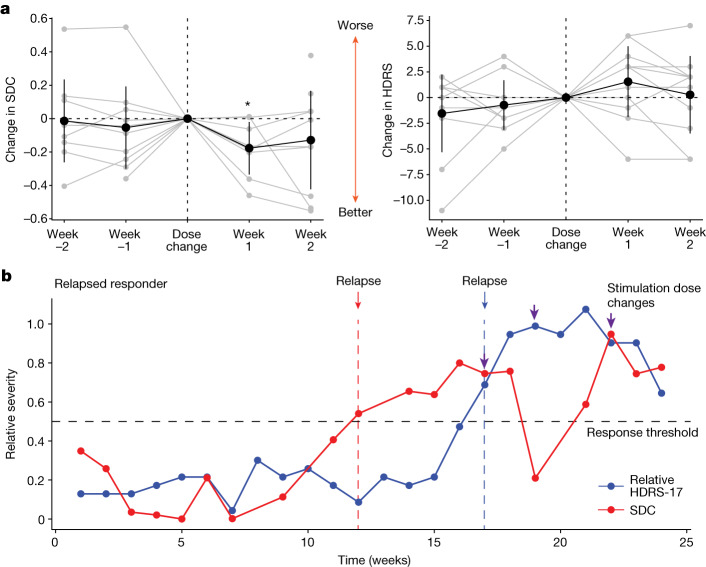
Response to stimulation change and validation in relapsed responder. **a**, Change in the SDC (left) and HDRS-17 (right) before and after the week of stimulation voltage change. Grey lines indicate the change relative to the week of a stimulation voltage change for each individual adjustment of stimulation voltage. Black lines indicate the average across all changes. Error bars indicate standard deviation (*n* = 8 stimulation dose changes). *P = 0.04 (one-sided Wilcoxon signed-rank test). **b**, Illustration of SDC in an out-of-sample participant who was a relapsed responder. The blue line denotes HDRS-17 and the red line denotes the SDC inferred from LFP features not used for training the classifier or SDC. The SDC increased above the threshold of 0.5 (grey dashed line) indicating relapse (red arrow) at week 12, indicating the relapse 5 weeks before it was observed in the HDRS-17 at week 17 (blue arrow). Purple arrows indicate changes in stimulation voltage levels. Note that stimulation voltage change did result in an SDC decrease as shown in **a**; however, the SDC did not stabilize until three stimulation voltage changes were made. The final voltage in this patient (4.5 V) was comparable with the average voltage in the typical responders (4.4 ± 0.57 V).

While all participants start at the same dose (a stimulation voltage of 3.5 V) at the beginning of the 24-week treatment protocol (except P001, who started at 3 V), the dose may be changed as deemed necessary by the study psychiatrist in increments of 0.5 V (Extended Data Table 1). The weeks in which these changes were made varied across participants (range 4–22 weeks after the beginning of therapeutic stimulation). This provides an opportunity to examine whether the SDC is affected by DBS dose adjustments. We found that increases in stimulation voltage resulted in a decrease in the SDC (that is, the LFP indicated progress towards the ‘well’ state) in the subsequent week (Fig. 3a left; −0.177 ± 0.111, one sample Wilcoxon signed-rank sum test *P* = 0.039), suggesting that the LFP features that capture stable depression recovery are affected by stimulation voltage change. By contrast, the changes in voltage did not result in a consistent or significant change in HDRS-17 scores in the subsequent week (Fig. 3a right, one sample Wilcoxon signed-rank test *P* = 0.151). We also found that the changes observed 1 week after the stimulation voltage change were statistically different from those observed 1 week after a random week when no stimulation voltage change was made (*P* = 0.034) using a shuffle-based procedure.

## SDC tracks relapse in held-out patient

To demonstrate the potential utility of the SDC in a clinical setting, we retrospectively analysed LFP data from one participant (P001) whose data were not included in training the classifier or the GCE. Thus, this participant served as an out-of-sample validation data point for the SDC as a depression state biomarker. P001 experienced a clinical relapse after 4 months in remission. P001 started the active stimulation phase with low HDRS-17 scores (less than 8) and had a sudden and sustained worsening of symptoms such that they were deemed a non-responder by week 16 (Fig. 3b blue line). Using the SDC trained on the five typical responders (but not trained on P001), the SDC correctly captures this trend in P001 by indicating a response state followed by a sick state (Fig. 3b red line). Interestingly, the SDC indicated a relapse from the brain signal (Fig. 3b red arrow) over 1 month before the clinical relapse measured by the HDRS-17 (Fig. 3b blue arrow), demonstrating that the brain biomarker could have predicted an impending instability and the need for earlier intervention before it was clinically apparent. In addition, dose increases (Fig. 3b purple arrows) resulted in decreases in SDC, but the effect did not persist until changes were made three times. Notably, the final stable dose in this patient (4.5 V) after the 6-month study period was comparable with the average dose in the typical responders.

To demonstrate the similarity between HDRS-17 and the SDC in this out-of-sample participant, we compared the states indicated by HDRS-17 and the SDC. As the therapeutic response was at the beginning of the observation phase, it is not possible to use the criteria described above for ‘stable response’. Yet, if we consider the two states as ‘sick’ and ‘response’ denoting a change in HDRS-17 of less than 50% decrease and greater than 50% decrease (respectively), we find that the SDC state accurately predicts the HDRS state 75% of the time over the 24-week treatment course (*P* = 0.029, shuffle-based procedure).

## White matter abnormality correlates of transition

**Fig. 4:**
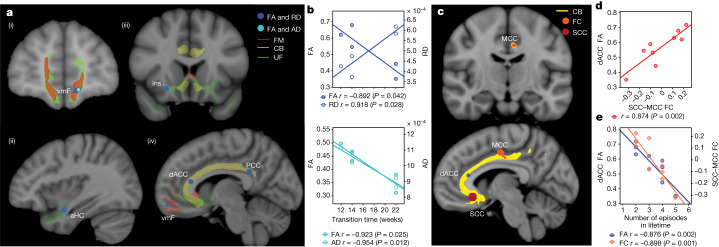
Structural and functional imaging correlates of transition to stable response. **a**, Regions showing correlation (Spearman’s rho) between the transition week and the white matter microstructure damage (*P* < 0.05), measured by both FA and radial diffusivity or FA and axial diffusivity, in vmF (i), aHC (ii), Ins (iii) and dACC and PCC (iv). **b**, Significant correlations (Spearman’s rho) were observed between the transition to ‘stable response’ week and FA (*P* = 0.042) and radial diffusivity (*P* = 0.028) in dACC (top) and FA (*P* = 0.025) and axial diffusivity (*P* = 0.012) in vmF (bottom). **c**, A significant correlation of dACC FA and functional connectivity between SCC and MCC with the number of episodes in a lifetime using all nine participants (excluding one participant because of artefact, Spearman’s rho *P* < 0.05) indicated by an orange dot in the coronal section (top). These regions are directly connected from the stimulation target via the cingulum bundle (bottom, yellow lines) which also contains the FA and radial diffusivity abnormality described in **a**. **d**, Post hoc correlation between FA and functional connectivity indicates a significant relationship between FA in the dACC and functional connectivity of the SCC and MCC (Spearman’s rho *P* = 0.002). **e**, Correlation (Spearman’s rho) between the number of episodes in a lifetime and functional connectivity of SCC and MCC (*P* = 0.001) and FA (*P* = 0.002).

Previous studies have shown that incomplete white matter pathway activation affects therapeutic outcomes in SCC DBS^11,16^. We hypothesized here that functional and structural abnormalities in these prespecified targeted white matter bundles may also influence the recovery trajectory, as inferred from the SDC. Using preoperative imaging, we found significant negative correlations between the weeks of transition to ‘stable response’ as identified from the SDC and white matter integrity, as indexed by both fractional anisotropy (FA) and radial diffusivity or FA and axial diffusivity. Regions having particularly significant correlations between structural integrity and time to recovery within the target network include the forceps minor, uncinate fasciculus, frontal–subcortical and cingulum bundles connecting the DBS target site to the ventromedial frontal cortex (vmF), anterior hippocampus (aHc), insular (Ins) and dorsal anterior and posterior cingulate cortex (dACC and PCC), respectively (Fig. 4a,b). These findings suggest that white matter microstructure alterations within the underlying targeted brain network results in longer DBS treatment times to achieve a stable response. Specifically, the radial diffusivity correlation with time to recovery provides evidence of baseline demyelination being a primary contributor to the white matter deficits that account for the variable time to recovery in patients.

In addition to a relationship between the stable SDC response time and white matter damage, we found a significant correlation of white matter abnormalities in the dACC to functional connectivity between the SCC and the midcingulate cortex (MCC) (*P* < 0.05) (Fig. 4c,d). This correspondence indicates a relationship between functional properties within the target network and structural properties that account for a prospective notion of disease severity as indexed by time to recovery. Furthermore, when considering the whole cohort, we found a significant negative correlation of both dACC FA and SCC–MCC functional connectivity with the number of lifetime depressive episodes experienced by each individual prior to SCC DBS (*n* = 9 participants; one excluded because of image artefact) (*P* < 0.05) (Fig. 4e). This concordance suggests that structural and functional deficits in the target network are also related to a retrospective notion of disease severity as indexed by the individual patient’s history of chronic depression.

## SDC tracks changes in facial expressions

**Fig. 5:**
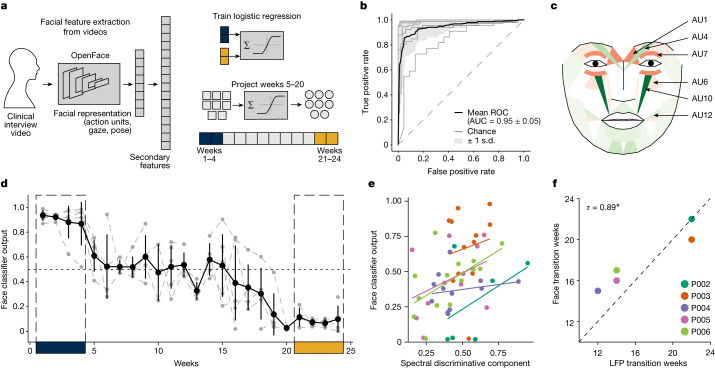
Facial expression correlates of SDC and transition times. **a**, Overview of facial expression classifier analysis. Facial landmarks are extracted from each frame of videos of clinical interviews and facial representation features (action units, gaze and pose) are estimated for each frame. Separate logistic regression classifiers are trained for each individual participant’s features to classify ‘sick’ and ‘stable response’ states. The features from the intermediate period (weeks 5−20) for each participant are then projected through the corresponding trained classifiers to get a prediction probability that serves as a measure of behavioural state. **b**, ROC curves of face classifier in classifying ‘sick’ and ‘stable response’ states within individual participants. Grey lines indicate the mean ROC curve of individual participants. Black lines indicate the mean ROC curve across participants. **c**, Muscle heat map output from Py-Feat showing consensus changes in action unit intensities between the ‘sick’ and ‘stable response’ states across all participants. Red colour indicates increases, while green colour indicates decreases. **d**, Trajectories of face classifier output for five typical responders. Grey lines indicate individuals and the black line indicates the average. Error bars indicate standard deviation (*n* = 5 participants). **e**, SDC versus face classifier output from weeks 5 to 20 for the five typical responders. Dots indicate weeks for individual participants, and the line indicates least-square fit regression. **f**, Correlation (Kendall’s tau) between transition weeks inferred from the SDC and face classifier output. Dots indicate individual participants. *P = 0.037.

In addition to standardized clinical rating scales, we quantified behavioural improvement using changes in facial expression extracted from videos of weekly clinical interviews. The features comprised summary measures of facial movements, including facial action units, eye gaze and head pose (Fig. 5a). Importantly, the features were not designed to explicitly relate to specific emotion constructs (for example, sadness). Similar to the LFP analyses (but entirely independent of the LFP data), we aimed to identify differences between the ‘sick’ and ‘stable response’ states using facial features. As there are considerable interindividual differences in facial expressions independent of depression, we used an individualized classifier for each patient to distinguish ‘sick’ and ‘stable response’ periods (in contrast to the single LFP classifier derived for the whole cohort). Random forest classifiers of facial expression features were able to classify ‘sick’ and ‘stable response’ states in each individual participant separately (AUROC 0.95 ± 0.05), suggesting that there are individualized yet consistent differences between the ‘sick’ and ‘stable response’ states (Fig. 5b). While we found a common set of distinct features (action units 1 and 7 and pose) across all participants in the consensus map (Fig. 5c), there were also many features that distinguished ‘sick’ and ‘stable response’ states unique to each participant.

**Extended Data Fig. 6:**
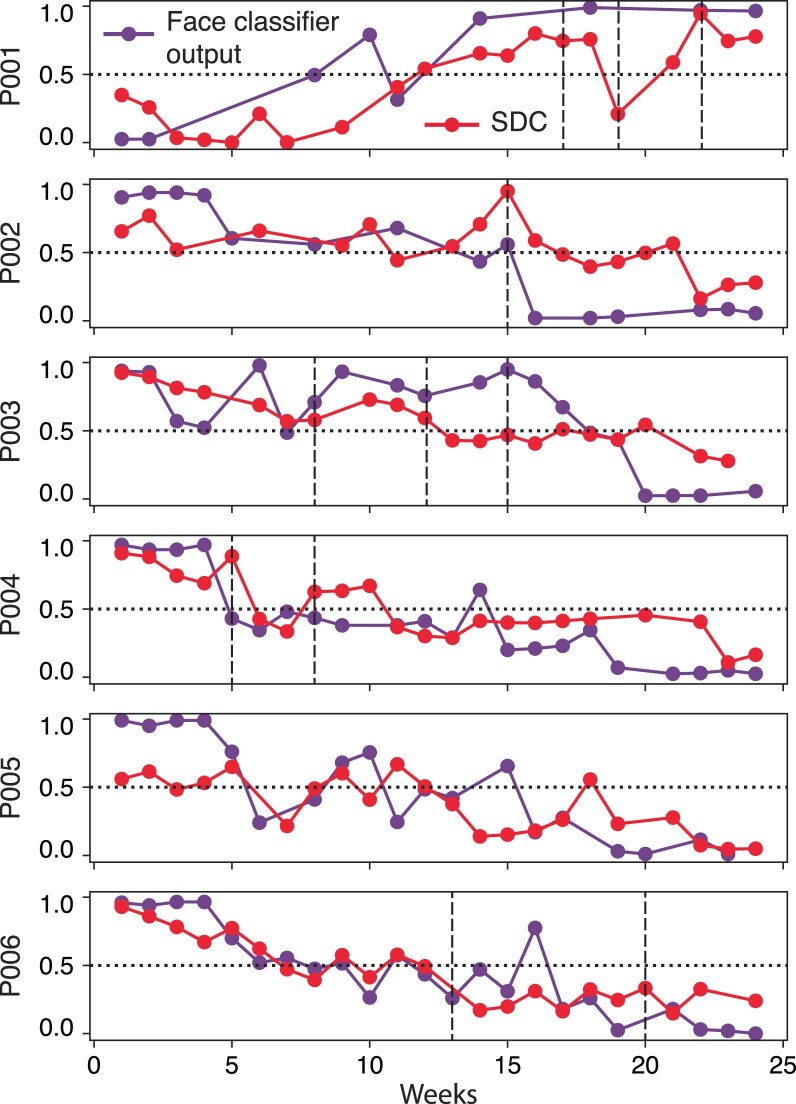
Trajectories of the SDC and face classifier output. Trajectory of face classifier output and spectral discriminative component for individual participants. P001 is the relapsed responder. The vertical dotted line indicates the week when the stimulation voltage was increased.

We then used these individual facial expression features extracted in the intermediate period (weeks 5−20) to obtain the classifier’s prediction of the disease state, which we termed face classifier output. As a secondary confirmation of the SDC biomarker, we compared the face classifier output with the SDC for each individual patient. We observed that the face classifier output’s trajectory is both qualitatively similar to the corresponding participant’s SDC trajectory (Fig. 5d; Extended Data Fig. 6), and quantitatively we found a significant relationship between the face classifier output and the SDC (Fig. 5e; linear mixed model, *F*(1.00, 51.74) = 6.54, *P* = 0.01). Next, we tested whether the face classifier output captures the changes from ‘sick’ to ‘stable response’ observed in the SDC. The face classifier output and the SDC have the same normalized scale (unlike HDRS-17), meaning they are directly comparable. Using a strict threshold (0.35) to binarize these measures for direct comparison, we found that the transition weeks from the ‘sick’ state to the ‘stable response’ state inferred from the SDC and the face classifier output were concordant (Fig. 5f; Kendall’s tau = 0.89, *P* = 0.037). Taken together, these results suggest that the SDC also accurately tracks changes in facial expressions accompanying recovery from depression.

## Discussion

In this study investigating long-term multimodal changes with SCC DBS, we derived the SDC as a common objective biomarker that accurately captured clinically defined ‘sick’ and ‘stable response’ states in all patients, as well as responding to changes in DBS stimulation. In addition, the transition to reach the ‘stable response’ state identified from the SDC was correlated with structural and functional irregularities in the targeted white matter tracts and was further concordant with a data-driven analysis of complex facial expressions. While this cohort experienced typical moment-to-moment mood variations as well as short-lived (experiential and LFP) effects with initial DBS exposure^15,17,18^, the SDC behaviour newly described here uniquely matches the clinical observation that sustained stable recovery requires weeks of ongoing chronic stimulation^19^.

**Extended Data Fig. 7:**
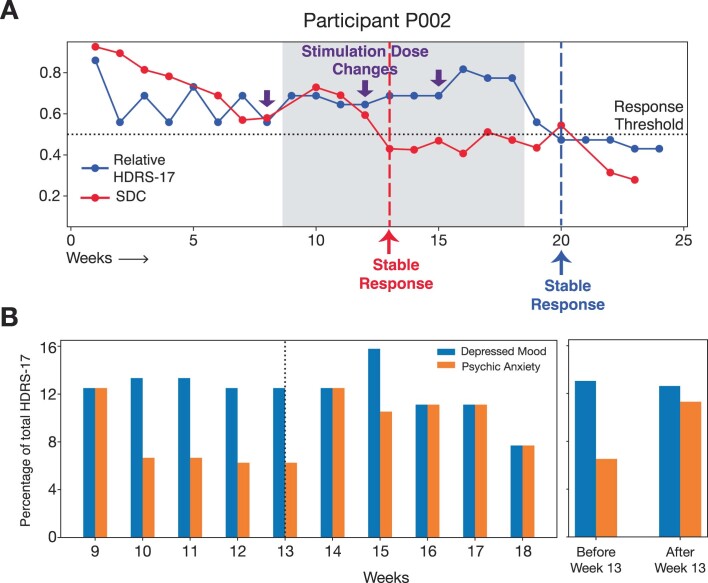
Case study of SDC indicating response while HDRS-17 indicates non-response. **a**, We observed in participant P002 SDC (red line) indicated stable response, defined using the criteria in Fig. 2e, at Week 13 while HDRS-17 (blue line) indicated ‘stable response’ at week 20. Stimulation voltage change (purple arrows) did not decrease HDRS-17 but decreased SDC. **b**, Psychic anxiety item (orange) of HDRS-17 increased contribution to total HDRS-17 while depressed mood (blue) remained constant suggesting the elevated HDRS-17 beyond week 13 (when SDC indicated stable response) may have been sustained by an increase in anxiety. Clinical notes support this hypothesis: “Biggest treatment issue is an internal resistance to the loss of depression and fears about what that means for her, including confronting feelings of loneliness and emptiness”.

Notably, the post hoc analysis of the relapsed responder demonstrates the potential value of the SDC in a clinical setting. Specifically, the SDC predicted the relapse approximately 5 weeks before structured interviews indicated the pending clinical change. Conversely, we also observed a different participant (P003) where the SDC indicated a transition to stable recovery well before the HDRS-17. Further analysis of the individual HDRS-17 items revealed that the apparent mismatch of the HDRS-17 and SDC was because of increasing anxiety symptoms without changes in core depression symptoms, a dissociation confirmed by clinical notes made by the study psychiatrist (Extended Data Fig. 7). Thus, our observations suggest that the derived SDC can aid in distinguishing the two scenarios laid out in Fig. 1e, adding critical information to inform rational clinical management decisions. To facilitate scalability, we also note that this biomarker is common across participants and does not require the individualization recently proposed in other strategies^20,21^. Replication in an independent cohort will provide additional specificity and sensitivity necessary for implementing a ‘clinician-in-the-loop’ DBS approach.

Multiple previous studies have also identified prominent (but not exclusive) beta band changes in acute SCC LFP dynamics with short-term stimulation exposure^14,15^ or with resting-state or emotional-challenge experiments without stimulation^22–25^. For example, previous studies demonstrate decreases in beta band power after brief bilateral intraoperative SCC stimulation^14,15^, consistent with the decreases in beta band power observed within the first month of chronic stimulation in the current cohort. The eventual transition to an increase in beta band power after chronic stimulation suggests that sustained, antidepressant responses are distinct from transient behavioural stimulation effects and thus are probably mediated by different mechanisms, including stimulation-induced plasticity^26,27^. Our findings support the broader hypothesis that beta band activity signals the establishment and maintenance of a status quo cognitive state^28^. In this context, we posit that the early desynchronization of beta band activity may correspond to release from the depressive maladaptive state enabling more flexible behaviour (reflected by increased HDRS variability), followed by an increase in beta band activity signalling the return of a new homeostatic set point after adaptation to chronic DBS (corresponding to stable recovery)^28^. Beyond the SCC, beta band activity has emerged as an important marker of dysfunction across many studies investigating mood disorders, including intracranial recordings in humans^29,30^, non-invasive electroencephalogram^31^ and rodent models^32^. Of note, the different regions investigated in these studies constitute the targets of our treatment network^33^, suggesting that the beta band changes we observed may reflect network-wide changes across multiple regions.

The long-term effects observed here with chronic DBS resemble the effects of slower-acting antidepressants, particularly selective serotonin reuptake inhibitors (SSRIs). The effect of SSRIs on 5-hydroxytryptamine neural activity in the dorsal raphe nucleus (DRN, one of the downstream targets of SCC DBS) has been shown to change over time, with acute suppression followed by restoration over 2 weeks (ref. ^34^). Interestingly, chronic DBS has been shown to act on DRN neurones, restoring serotonergic pathways from DRN to limbic regions that include the vmF^35^.

While all patients were implanted to affect the same four white matter bundles, the degree of increased radial diffusivity and decreased FA (typically suggesting demyelination) within this target network was correlated with longer recovery times. Further supporting the role of region-specific white matter integrity in depression pathophysiology is complementary postmortem findings in TRD suicides, which identify local myelin and oligodendroglia abnormalities in and around the SCC region and its projections^36–38^. Furthermore, dACC FA is significantly associated with functional connectivity deficits between the stimulation target and MCC, which are directly connected via the cingulum bundle^33,39^. Importantly, our finding of a negative correlation between white matter deficits and the number of lifetime depressive episodes is consistent with a large depression cohort study that reported lower FA and higher radial diffusivity with recurrent patients compared with single-episode patients, as well as previous studies relating the cumulative effects of depressive episodes on brain microstructure^40^. Network reorganization may be a potential mechanism of the transition from acute to chronic response with SCC DBS, consistent with animal studies suggesting that chronic stimulation may lead to neuroplastic changes, resulting in remyelination of targeted tracts^26,41^ or engagement of homeostatic plasticity mechanisms to produce long-term changes^42^. The availability of new magnetic resonance imaging (MRI)-compatible DBS IPGs will enable direct measurements of structural and functional connectivity changes within the stimulated network over time to test these hypotheses.

A patient’s appearance is a core component of a physician’s clinical assessment during diagnosis and recovery, and our personalized facial expression analysis of depression states provides a robust independent readout of these clinical impressions that concords with the SDC. While there was a clear overlap of the face action units (AUs) that changed across participants, the inability to derive a single sick/well classifier (either due to inherent variability or small sample size limitations) meant that the model could only be used as a descriptive tool instead of a prospective estimate of the current depression state. The common changes across participants do involve AUs previously linked to classic constructs of both sadness and happiness^43^, as well as the electromyography patterns of pain and despair defined by Duchenne and Darwin in the 1860s^44,45^. Importantly, stimulation of the cingulum bundle directly affects dACC projections to the facial nuclei that innervate the eyes and upper face (for example, orbicularis oculi muscle and frontalis/corrugator muscular complex)^46^ that dominate the AU changes seen across participants^46^. Deficits in this pattern of facial movement (loss of mimetic facial expression with preservation of volitional movement) are well described with cingulum bundle lesions^47^, and the dACC white matter lesion reported above is adjacent to the cingulate face region^48,49^. While the upper face does not work in isolation, the symmetric, bilateral change pattern across patients is consistent with the normalization of emotional rather than volitional facial movement (moderated by M1 projections from the lateral cortex, which is a region not impacted directly by SCC DBS).

The current study has several limitations. First, the LFP analysis here is limited to six of ten participants due to prototype device challenges (that is, data artefacts and protocol changes after pilot implantations). Nonetheless, the derived SDC biomarker was reliable across all participants, including the held-out participant. Second, the results presented are from LFP collected with therapeutic stimulation temporarily turned OFF to eliminate significant stimulation-related artefacts^50^. However, while there is practical convenience to estimating a biomarker without interrupting therapeutic stimulation, the lack of negative clinical effects associated with relatively short SCC DBS discontinuation makes it feasible to calculate this biomarker during transient periods without the technical confound. Third, we have not explicitly modelled acute moment-to-moment distress^20,30^, which would validate the specificity and potentially enhance the behavioural interpretability of our chronic biomarker. Future studies with increased data collection frequency will allow the modelling of potential LFP signatures of transient mood or anxiety symptoms. Finally, the analysis here is retrospective, leaving open questions about the exact use of the SDC in determining the precise timing of optimal stimulation adjustments or the introduction of adjunct rehabilitative interventions such as cognitive behavioural therapy or mindfulness training.

## Methods

### Participants and clinical assessments

Ten participants with treatment-resistant major depressive disorder were consecutively enrolled in a single-site clinical trial with a single active DBS interventional arm using a prototype DBS device that allowed the collection of local field potentials from the stimulation site (ClinicalTrials.gov identifier NCT01984710). Participant characteristics are provided in Extended Data Table 1. All patients provided written informed consent to participate in the study. The protocol was in accordance with the Declaration of Helsinki. The protocol was approved by the Institutional Review Boards at Emory University, Georgia Institute of Technology and the Icahn School of Medicine, and the US Food and Drug Administration under a physician-sponsored Investigational Device Exemption (IDE G130107) and was monitored by the Emory University Department of Psychiatry and Behavioral Sciences Data and Safety Monitoring Board. Clinical symptom severity was assessed by an independent rater using the 17-item HDRS, MADRS and self-reported Beck Depression Inventory during weekly visits to the laboratory, among other behavioural scales. Patients met weekly with the study psychiatrist, who could make stimulation adjustments (increasing voltage by 0.5 V bilaterally) using a combination of HDRS-17 changes relative to the previous week and their clinical judgement. Following established criteria, a decrease in HDRS-17 scores greater than 50% of the presurgical average was set as the threshold for ‘response’. Remission was defined as HDRS-17 < 8 and MADRS < 10. Relative HDRS-17 and relative MADRS were computed as proportions of the presurgical average of HDRS-17 and MADRS, respectively.

We report the analysis of LFPs from six participants listed in Extended Data Table 1 during a period of 6 months from the initiation of DBS therapy. Two participants were excluded from the analysis, as they had LFP data distorted by an amplifier clipping artefact (one participant) or heartbeat artefacts (one participant). Both these participants were responders (more than 50% decrease in HDRS-17 from presurgical baseline), and one of them achieved remission (HDRS-17 < 8). The weekly trajectories of the excluded participants were not qualitatively different from the participants included in the study, as shown in Extended Data Fig. 2c.

### SCC DBS and dose adjustment

Bilateral electrode array leads (3387, Medtronic) were implanted in each participant, one in each SCC (Fig. 1a) as determined from tractography previously described in Riva-Posse et al.^12^ A connectome-based targeting approach was used to identify targets that intersect four white matter pathways: forceps minor, cingulum bundle, uncinate fasciculus, and frontostriatal fibres (Fig. 1b). Stimulation was delivered using a voltage-controlled pulse generator Activa PC + S which also served as the local field potential acquisition system (Medtronic). DBS therapy started at least 30 days after the implantation surgery to allow for recovery from surgery. Therapy consisted of bilateral monopolar stimulation on a single contact per hemisphere at 130 Hz with 90 µs pulse width. Stimulation amplitude was initially set at 3.5 V for all participants except P001. The initial amplitude for P001 was set at 3.0 V, as the participant’s symptoms were below the remission threshold at the beginning of the observation phase. During the observation phase, location, pulse width, and stimulation frequency remained unchanged. Dose was increased in steps of 0.5 V at unspecified intervals based on the study clinician’s (P.R.-P./A.C.) assessment of patient progress as described above. The initial stimulation voltage, stimulation voltage at the end of the 6-month study period and number of times stimulation voltage was changed in each participant are listed in Extended Data Table 1. None of the participants needed a stimulation dose decrease.

### LFP recordings and feature extraction

Local field potentials were acquired at a sampling rate of 422 Hz using the Medtronic Activa PC + S system^51^ (Medtronic Activa PC + S 8180 Sensing Software) performing a differential recording from electrode contacts on either side of the stimulation contact to allow for common-mode rejection of noise, as well as stimulation-related artefacts. LFPs were acquired weekly during the observation phase in a single 15-min session in the laboratory. Each session consisted of two segments of approximately 7.5 min each: one with stimulation turned on, and the other with stimulation turned off. Only the segments with stimulation turned off were included in the analysis, as the presence of stimulation-related artefacts precluded functional connectivity and cross-frequency coupling analyses. The first 10 s of the stimulation-off period was discarded due to the presence of stimulation offset artefact (a slowly decaying signal reaching baseline). In addition, periods during which amplifier switching artefacts (presence of spike-like artefacts) were present were discarded. Finally, device-related frequency-drift artefacts were observed in the beta and gamma bands in a subset of the recordings of some participants. A robust principal component analysis approach separated the device-related artefact into sparse components, while the low-rank component contained the neural signals and was used in further analysis.

All LFP analyses were performed using custom-written scripts in Python (v.3.6) and Matlab (R2018b). LFP recorded within a session was divided into 10-second segments from which spectral power, coherence and phase-amplitude coupling (PAC) were estimated. Spectral power and magnitude-squared coherence were estimated using the Python library Nitime’s^52^ (v.0.9) multi-taper fast Fourier transform approach with an adaptive procedure for setting the weights of tapers. Spectral power and coherence in canonical frequency bands (delta: 1–4 Hz; theta: 4–8 Hz; alpha: 8–13 Hz; low beta: 13–20 Hz; high beta: 20–30 Hz; gamma: 30–40 Hz) were then extracted as features for classification. The upper limit of the gamma band was restricted to 40 Hz instead of 50 Hz because of the presence of device-related artefacts in the range of 40–50 Hz.

PAC was estimated using the PACtools (v.0.3.1) python library^53^. The algorithm described in work by Tort et al.^54^ was used to compute the coupling between low frequency (1–15 Hz) phase and high-frequency (15–45 Hz) amplitude. Comodulograms were visually inspected to identify PAC regions of interest, and PAC values between the delta band (1.5–3.0 Hz) and the high beta/gamma band (20–35 Hz) were extracted as features. This procedure was followed to restrict the dimensionality of the features for the classifier, as including all the possible interactions would have considerably increased the feature set size. Thus, the overall dimensionality of the feature set was 20 (six spectral features per hemisphere, six coherence features and one PAC feature per hemisphere).

### LFP classification and inferring SDC

Neural network models were used to classify LFP features using PyTorch^55^ (v.1.11.0). The parameters for the neural network models are listed in Extended Data Table 2. LFP spectral features were individually scaled between 0 and 1 as a preprocessing step. A fivefold leave-one-out cross-validation was performed at the participant level to ensure generalizability. Models were fitted using LFP features from four out of five participants, while the features from the fifth participant served as the test set. This procedure was repeated five times such that features from all five participants served as a test case.

We use the GCE framework^13^ to identify interpretable features in the data determinative of the classifier’s output. Conceptually, GCE can be thought of as a form of dimensionality reduction in which only a subset of the low-dimensional representation has a causal effect on the classifier output (Fig. 1d). This partitioning of the low-dimensional representation into classifier-relevant and classifier-irrelevant dimensions is accomplished by augmenting the objective of an auto-encoder with a mutual information term that encourages a portion of the low-dimensional representation to influence the classifier output^13^. We call the subset of dimensions in the low-dimensional representation relevant to the classifier’s output the ‘discriminative components’ and the subset of the dimensions that contribute to representing the data but do not affect the classifier’s output the ‘non-discriminative components’.

In the present work, the GCE was implemented using two separate networks: a feature compression network that maps the data from the feature space to the low-dimensional latent space and a feature reconstruction network that reconstructs the feature space data from the latent components (Fig. 1d). The low-dimensional latent components were termed the SDCs in one dimension and spectral non-discriminative components (SNDCs) in the remaining dimensions, based on the choice of parameters of GCE. The networks were trained to maximize the similarity of the reconstructed data and the true data using a loss function commonly used in variational auto-encoders^56^, as well as the information flow from the SDC to classifier output using a loss function developed in ref. ^13^. The GCE was trained with features extracted from LFP collected during the first month and last month of therapy in all participants and a classifier trained on the same data. Information flow from discriminative components to classifier output was higher than that of non-discriminative components, indicating that the SDC captures the features that determine the classifier output (Extended Data Fig. 2a). Leave-one-out cross-validation was performed to make sure the model did not overfit. In brief, GCE was trained on four out of the five participants and used to reconstruct the data of the fifth participant, which was then used to evaluate the classifier’s performance. This procedure was repeated five times, leaving a unique participant’s data out in each fold. The classifier’s performance was comparable with the original data (Extended Data Fig. 2b). In addition, to verify whether only the discriminative component affected the classifier prediction, one of the components was randomized, with other components unaffected, and the classifier performance on the reconstructed data was evaluated. The entire procedure was performed in a leave-one-out fashion, as described before. The performance of the classifier was affected when the discriminative component was randomized but not when the non-discriminative components were randomized, verifying our design requirements. The reconstruction performance was evaluated by (1) verifying that the classification performance of a neural network classifier trained on the reconstructed data matched the performance of the classifier trained on the original data and (2) training a separate neural network classifier with original data and testing on the reconstructed data. In both cases, the performance of the classifiers was comparable with the original classifier (case (i) AUC = 0.8; case (ii) AUC = 0.89 ± 0.03; Extended Data Fig. 2c) suggesting the reconstruction captured the salient features of the original data. The parameters of the networks are listed in Extended Data Table 2. A summary of the training and testing datasets used for different models is listed in Extended Data Table 3.

The trained feature compression network was used to infer discriminative components of the LFP collected during months 2–5. LFP spectral features, computed in 10-second segments, were minimum–maximum scaled to the training set (LFP features from months 1 and 6) and projected through the feature compression network to infer discriminative and non-discriminative components. The SDC was transformed to the probability of belonging to the ‘sick’ state which was determined as the ratio of the number of SDC values greater than or equal to SDC from the ‘sick’ state weeks to the total number of SDC values (equation below). This allowed the SDC to be compared directly against the face classifier output. The SDC was then averaged across the 10-second segments within a week:

$${P}_{{\rm{s}}{\rm{d}}{\rm{c}}}=n({\rm{S}}{\rm{D}}{\rm{C}}\ge {{\rm{S}}{\rm{D}}{\rm{C}}}_{{\rm{s}}{\rm{i}}{\rm{c}}{\rm{k}}})/(n({{\rm{S}}{\rm{D}}{\rm{C}}}_{{\rm{s}}{\rm{i}}{\rm{c}}{\rm{k}}})+n({{\rm{S}}{\rm{D}}{\rm{C}}}_{\text{stable response}}))$$

To map what features correspond to the SDC and SNDCs, the component values were varied in the latent space and passed through the feature reconstruction network. The resulting changes in the features were fit with second-order polynomials, and the magnitude of the coefficients served as an indicator of feature change between weeks 1–4 and weeks 21–24. As the slope term captured most of the change, it was used as a measure of the features underlying the SDC.

### Transition to stable response

Patients receiving chronic therapeutic SCC DBS have been observed to show a characteristic response trajectory marked by a transient period of increased behavioural reactivity and instability followed by an improvement in symptoms that is sustained and stable^3^. We inferred the week at which each participant reached this ‘stable response’ state based on weekly changes in HDRS-17, the SDC or the face classifier output (Fig. 2e). The transition was defined as the first of two consecutive weeks when the participant’s measure fell below a defined threshold and did not increase beyond the threshold for two or more weeks.

In the case of HDRS-17, the relative score, which is the ratio of the aggregate score to the average of the presurgical baseline scores, was used to define the states. A threshold of 0.5, indicating a decrease of 50% from the presurgical baseline, was used to follow the widely accepted definition of clinical response. In the case of the SDC and the face classifier output, it is not clear what the exact thresholds that correspond to clinical response should be. Therefore we used the ROC curve, which focuses on sensitivity and selectivity of discriminability instead of hard thresholds, to compare against HDRS-17. However, when compared against each other, it is possible to use the same thresholds, as the values indicate the probability of being in the ‘sick’ state. We used a more conservative threshold of 0.35 to identify the transition to ‘stable response’.

The concordance between the weeks of transition was evaluated using Kendall’s tau metric, which is a rank-based correlation measure. Kendall’s tau reflects the similarity in the ranks of the transition weeks, that is, do the participants who exhibit a transition in SDC early also exhibit a transition in the face early and vice versa?

### Image acquisition and processing

High-resolution structural T1-weighted (T1w), resting-state functional MRI (fMRI), and diffusion-weighted images (DWIs) were acquired on a 3 T Siemens Tim Trio and Prisma MRI scanner (Siemens Medical Solutions). T1w images were collected using a three-dimensional magnetization-prepared rapid gradient–echo (MPRAGE) sequence with the following parameters: sagittal slice orientation; resolution = 1.0 mm × 1.0 mm × 1.0 mm; repetition time (TR) = 2,600 ms; inversion time (TI) = 900 ms; echo time (TE) = 3.02 ms; flip angle = 8°. Resting-state fMRI was performed with patients’ eyes open for 7.4 min using two different scanners: (1) Tim Trio (*n* = 6), a Z-SAGA sequence, to recover areas affected by susceptibility artefacts; 150 volumes; 30 axial slices; voxel size = 3.4 × 3.4 × 4 mm^3^; matrix = 64 × 64; TR = 2,950 ms; TE = 30 ms and (2) Prisma (*n* = 4), 460 volumes; 56 axial slices; voxel size = 2 × 2 × 2 mm^3^; matrix = 110 × 110; TR = 1,000 ms; TE = 30 ms. DWIs were acquired using single-shot spin-echo echo-planar imaging sequence with the following parameters: 64 non-collinear directions with five non-DWIs (b0), *b* value = 1,000 s mm^−^^2^; number of slices = 64; field of view = 256 × 256 mm^2^; voxel size = 2 × 2 × 2 mm^3^; TR = 11300 ms; TE = 90 ms. Additional full DWI dataset with opposite phase encoding was also collected to compensate for the susceptibility-induced distortion.

All images were preprocessed using the FMRIB Software Library (FSL; http://www.fmrib.ox.ac.uk/fsl/)^57^ (v.6.0) and Analysis of Functional NeuroImages (AFNI, http://afni.niml.nih.gov/afni/) software (v.23.1.06). The T1w image was skull stripped and normalized to the MNI152 template using the fsl_anat toolbox. The standard preprocessing pipeline, including de-spiked and corrected for slice time acquisition differences and head motion, implemented in the AFNI was used for resting-state fMRI preprocessing. The remaining effect of noise signals, including head motion inferences, signals from the CSF, and local white matter, were removed. Subsequently, the data were band-pass filtered (0.01 < *f* < 0.1 Hz) and spatially smoothed up to 8 mm full-width at half-maximum (FWHM) using 3dBlurToFWHM in AFNI. The preprocessed fMRI data were normalized to the MNI152 template using previously generated T1w normalization warp fields. The mean time series of the bilateral SCC seed (±6, +24, −11)^58^ was correlated voxel-wise with the rest of the brain. The voxel-wise correlation coefficient maps were then converted to *Z* scores by Fisher’s *r*-to-*z* transformation. The *Z* score determined the level of functional connectivity of the SCC seed. DWI data underwent distortion and motion collection using the Eddy toolbox and a local tensor fitting to calculate the FA map. Tract-Based Spatial Statistics processing was performed for group analysis^59^. In brief, individual FA images were aligned to the standard FMRIB58 FA template using a nonlinear registration, and the normalized FA images were then averaged to create a mean FA image. The mean FA image was thinned to create a FA skeleton representing white matter tracts common to all patients. A threshold value of 0.2 was used to exclude adjacent grey matter or cerebrospinal fluid voxels. A similar process was performed for radial diffusivity and axial diffusivity.

A VTA was generated using the StimVison toolbox^60^ with patients’ specific chronic stimulation settings (that is, 130 Hz, 3.5 V, 90 μs). White matter tracts passing through VTA were extracted in each participant using the Xtract toolbox in FSL^61^ and then averaged to generate a white matter tract mask that represents the common activation pathways of all five participants. Three white matter masks, including forceps minor, cingulum bundle and uncinate fasciculus, were used for the statistical analysis. Within the specific tracks of the FA skeleton, Spearman’s rank correlation between white matter integrity measures (FA, radial diffusivity and axial diffusivity) and the inferred transition times were performed to evaluate whether white matter microstructure at baseline could predict the inferred transitions in states.

To further explore the relationship between altered white matter microstructures/abnormal brain activity and DBS recovery trajectory, post hoc correlation analyses were conducted in the identified brain regions from the correlation analysis of transition times with imaging using all nine responders. In brief, Spearman’s rank correlation analysis (age and sex controlled) was performed between baseline white matter integrity (FA) and depression clinical features, including depression severity (HDRS-17), duration of current episode, the number of episodes in a lifetime and length of illness (duration between onset and surgery). In addition, the same analyses were performed for the resting-state functional connectivity using the bilateral SCC seeds.

### Facial expression analysis

In addition to clinical assessments, behavioural changes were estimated from facial expressions extracted from weekly videos of participants collected during the weekly psychiatric clinical interviews where LFPs were recorded, and DBS management, including dose changes, was determined. Videos were recorded using a static, tripod-mounted video camera recording at 30 frames per second. The sessions lasted approximately 30 min.

Videos were partitioned into 5-min windows for feature generation, with the remainder discarded. Each window was processed with the OpenFace facial behaviour analysis toolkit v.2.0 (ref. ^62^). This open-source software produces presence, intensity and confidence estimations for 18 facial action units, eye gaze and head pose vectors, as well as 68 facial landmark positions for each frame. The 30 Hz frame rate was sufficiently granular to yield a temporal resolution to capture microexpressions (less than 0.5 second duration) as well as macroexpressions (0.5–4.0 s). Data from frames with less than 93% confidence were discarded. The most common reason for discarding frames was the obstruction of the participants’ faces by their hands. From these first-order features, we generated second-order features consisting of envelope metrics (mean, median, quantiles, skew, kurtosis, variance) and covariance between features. From gaze and pose vectors, we generated velocity, acceleration, jerk and their envelope metrics. This processing was implemented in Python resulting in 1,073 features overall.

Using the same rationale as for the LFP classification, the facial expression features most differentially expressed between the ‘sick’ (weeks 1–4) and ‘stable response’ (weeks 21−24) states were identified using a paired two-sided *t*-test and used as input to train binary classifiers for each participant. For unbalanced sample sets due to sparse recordings, SMOTE^63^ was used to oversample the minority class. A random forest classifier with tenfold cross-validation was implemented in the Python sklearn (v.1.1.1) library^64^ to discriminate the ‘sick’ from the ‘stable response’ state for each participant. Following this, the trained classifiers were evaluated on the samples from the intermediate period to get the probability of being in the ‘sick’ state. The classifier predictions were termed ‘face classifier outputs’ and served as another behavioural marker to track response during ongoing DBS. We used Py-Feat toolbox^65^ to visualize the changes in facial expression features between the sick and stable response states. Custom scripts incorporating Py-Feat functions were used to generate muscle heat maps of the changes in AU intensities.

### Statistical analysis

Hypothesis testing of changes in HDRS-17, SDC and individual features was performed using a one-sided Wilcoxon signed-rank test. The non-parametric test was chosen to account for the small sample size and inability to test for normality. The small sample size of the current study does not have sufficient power to test statistical significance at 0.05 in a two-sided test, even when the direction of changes is readily apparent. Therefore, we used a one-sided test with a threshold of 0.05 and also confirmed statistical significance in a two-sided test with a relaxed threshold of 0.1. Linear mixed models were used to test the association between the SDC and clinical assessment scores and the SDC and face classifier output (with the SDC as the fixed factor and participants as the random factor). Models were fitted using the ‘lmertest’ package^66^ (v.3.1.3), which uses a Sattherwaite approximation for degrees of freedom for ANOVA. The threshold was set at uncorrected *P* < 0.05 for all correlation analyses between imaging and the SDC.

### Reporting summary

Further information on research design is available in the Nature Portfolio Reporting Summary linked to this article.

## Online content

Any methods, additional references, Nature Portfolio reporting summaries, source data, extended data, supplementary information, acknowledgements, peer review information; details of author contributions and competing interests; and statements of data and code availability are available at 10.1038/s41586-023-06541-3.

### Supplementary information

Reporting Summary

## Figures and Tables

**Extended Data Table 1:**
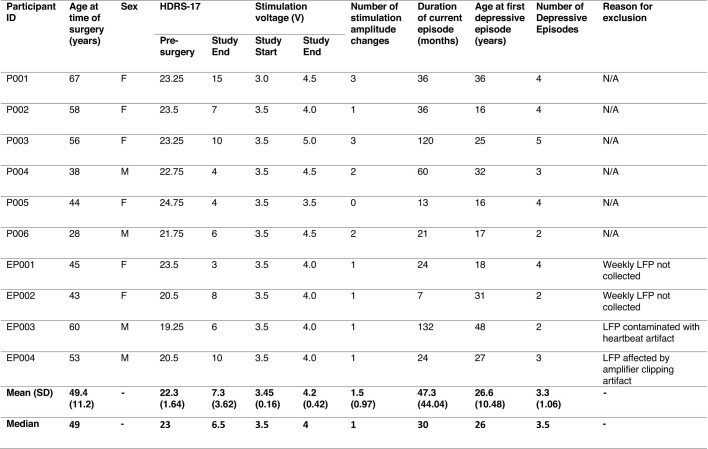
Overview of study participants’ demographics

**Extended Data Table 2:**
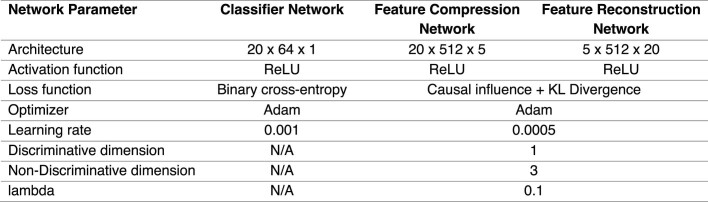
ML models parameters

Parameters of the neural network classifier and GCE models.

**Extended Data Table 3:**
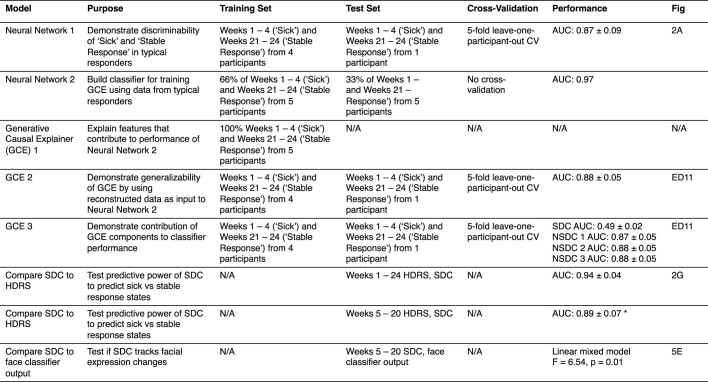
Summary of training and validation procedure for ML models

*Computed over four participants since the transition in HDRS for P006 was at week 5 and hence only one class was present in weeks 5–20. Since it is not possible to construct ROC curve without two classes, P006 was excluded.

## Data Availability

The data that support the findings of this study are publicly available via the Data Archive for The Brain Initiative (DABI) at https://dabi.loni.usc.edu/dsi/1UH3NS103550/UXUF7822Z3JL.

## Code availability

Custom code used in this work is available at https://dabi.loni.usc.edu/dsi/1UH3NS103550/UXUF7822Z3JL.

## References

[1] Crowell, A. L. et al. Long-term outcomes of subcallosal cingulate deep brain stimulation for treatment-resistant depression. *Am. J. Psychiatry*10.1176/appi.ajp.2019.18121427 (2019). 10.1176/appi.ajp.2019.18121427 31581800

[2] Holtzheimer PE (2017). Subcallosal cingulate deep brain stimulation for treatment-resistant depression: a multisite, randomised, sham-controlled trial. Lancet Psychiatry.

[3] Crowell AL, Garlow SJ, Riva-Posse P, Mayberg HS (2015). Characterizing the therapeutic response to deep brain stimulation for treatment-resistant depression: a single center long-term perspective. Front. Integr. Neurosci..

[4] Mayberg HS (2005). Deep brain stimulation for treatment-resistant depression. Neuron.

[5] Holtzheimer PE (2012). Subcallosal cingulate deep brain stimulation for treatment-resistant unipolar and bipolar depression. Arch. Gen. Psychiatry.

[6] Puigdemont D (2012). Deep brain stimulation of the subcallosal cingulate gyrus: further evidence in treatment-resistant major depression. Int. J. Neuropsychopharmacol..

[7] Lozano AM (2008). Subcallosal cingulate gyrus deep brain stimulation for treatment-resistant depression. Biol. Psychiatry.

[8] Ramasubbu, R., Lang, S. & Kiss, Z. H. T. Dosing of electrical parameters in deep brain stimulation (DBS) for intractable depression: a review of clinical studies. *Front. Psychiatry* **9**, 302 (2018). 10.3389/fpsyt.2018.00302 PMC6050377 30050474

[9] Urban EJ, Charles ST, Levine LJ, Almeida DM (2018). Depression history and memory bias for specific daily emotions. PLoS ONE.

[10] Solhan MB, Trull TJ, Jahng S, Wood PK (2009). Clinical assessment of affective instability: comparing EMA indices, questionnaire reports, and retrospective recall. Psychol. Assess..

[11] Riva-Posse P (2014). Defining critical white matter pathways mediating successful subcallosal cingulate deep brain stimulation for treatment-resistant depression. Biol. Psychiatry.

[12] Riva-Posse P (2018). A connectomic approach for subcallosal cingulate deep brain stimulation surgery: prospective targeting in treatment-resistant depression. Mol. Psychiatry.

[13] O’Shaughnessy M, Canal G, Connor M, Rozell C, Davenport M (2020). Generative causal explanations of black-box classifiers. Adv. Neural Inf. Process. Syst..

[14] Smart O (2018). Initial unilateral exposure to deep brain stimulation in treatment-resistant depression patients alters spectral power in the subcallosal cingulate. Front. Comput. Neurosci..

[15] Sendi MSE (2021). Intraoperative neural signals predict rapid antidepressant effects of deep brain stimulation. Transl. Psychiatry.

[16] Howell B (2019). Quantifying the axonal pathways directly stimulated in therapeutic subcallosal cingulate deep brain stimulation. Hum. Brain Mapp..

[17] Choi KS, Riva-Posse P, Gross RE, Mayberg HS (2015). Mapping the “depression switch” during intraoperative testing of subcallosal cingulate deep brain stimulation. JAMA Neurol..

[18] Ramasubbu R, Anderson S, Haffenden A, Chavda S, Kiss ZH (2013). Double-blind optimization of subcallosal cingulate deep brain stimulation for treatment-resistant depression: a pilot study. J. Psychiatry Neurosci. JPN.

[19] Dandekar MP, Fenoy AJ, Carvalho AF, Soares JC, Quevedo J (2018). Deep brain stimulation for treatment-resistant depression: an integrative review of preclinical and clinical findings and translational implications. Mol. Psychiatry.

[20] Scangos KW (2021). Closed-loop neuromodulation in an individual with treatment-resistant depression. Nat. Med..

[21] Sheth SA (2022). Deep brain stimulation for depression informed by intracranial recordings. Biol. Psychiatry.

[22] Clark DL, Brown EC, Ramasubbu R, Kiss ZHT (2016). Intrinsic local beta oscillations in the subgenual cingulate relate to depressive symptoms in treatment-resistant depression. Biol. Psychiatry.

[23] Huebl J (2016). Processing of emotional stimuli is reflected by modulations of beta band activity in the subgenual anterior cingulate cortex in patients with treatment resistant depression. Soc. Cogn. Affect. Neurosci..

[24] Merkl A (2016). Modulation of beta-band activity in the subgenual anterior cingulate cortex during emotional empathy in treatment-resistant depression. Cereb. Cortex.

[25] Neumann W-J (2014). Different patterns of local field potentials from limbic DBS targets in patients with major depressive and obsessive compulsive disorder. Mol. Psychiatry.

[26] Gibson EM (2014). Neuronal activity promotes oligodendrogenesis and adaptive myelination in the mammalian brain. Science.

[27] Ramirez-Mahaluf JP, Roxin A, Mayberg HS, Compte A (2017). A computational model of major depression: the role of glutamate dysfunction on cingulo-frontal network dynamics. Cereb. Cortex.

[28] Engel AK, Fries P (2010). Beta-band oscillations—signalling the status quo?. Curr. Opin. Neurobiol..

[29] Kirkby LA (2018). An amygdala-hippocampus subnetwork that encodes variation in human mood. Cell.

[30] Xiao J (2023). Decoding depression severity from intracranial neural activity. Biol. Psychiatry.

[31] Benschop L (2021). Electrophysiological scarring in remitted depressed patients: elevated EEG functional connectivity between the posterior cingulate cortex and the subgenual prefrontal cortex as a neural marker for rumination. J. Affect. Disord..

[32] Hultman R (2018). Brain-wide electrical spatiotemporal dynamics encode depression vulnerability. Cell.

[33] Heilbronner SR, Haber SN (2014). Frontal cortical and subcortical projections provide a basis for segmenting the cingulum bundle: implications for neuroimaging and psychiatric disorders. J. Neurosci..

[34] El Mansari M, Sánchez C, Chouvet G, Renaud B, Haddjeri N (2005). Effects of acute and long-term administration of escitalopram and citalopram on serotonin neurotransmission: an in vivo electrophysiological study in rat brain. Neuropsychopharmacology.

[35] Veerakumar A (2014). Antidepressant-like effects of cortical deep brain stimulation coincide with pro-neuroplastic adaptations of serotonin systems. Biol. Psychiatry.

[36] Rajkowska G (2015). Oligodendrocyte morphometry and expression of myelin-related mRNA in ventral prefrontal white matter in major depressive disorder. J. Psychiatr. Res..

[37] Sacchet MD, Gotlib IH (2017). Myelination of the brain in major depressive disorder: an in vivo quantitative magnetic resonance imaging study. Sci. Rep..

[38] Öngür D, Drevets WC, Price JL (1998). Glial reduction in the subgenual prefrontal cortex in mood disorders. Proc. Natl Acad. Sci..

[39] Kleckner IR (2017). Evidence for a large-scale brain system supporting allostasis and interoception in humans. Nat. Hum. Behav..

[40] van Velzen LS (2020). White matter disturbances in major depressive disorder: a coordinated analysis across 20 international cohorts in the ENIGMA MDD working group. Mol. Psychiatry.

[41] Bambico FR (2015). Neuroplasticity-dependent and -independent mechanisms of chronic deep brain stimulation in stressed rats. Transl. Psychiatry.

[42] Chai Z, Ma C, Jin X (2019). Homeostatic activity regulation as a mechanism underlying the effect of brain stimulation. Bioelectron. Med..

[43] Ekman, P., Friesen, W. V. & Ellsworth, P. *Emotion in the Human Face: Guidelines for Research and an Integration of Findings* (Pergamon Press, 1972).

[44] Duchenne, G.-B. *Mécanisme de la Physionomie Humaine ou Analyse Électro-physiologique de l’Expression des Passions* (Librairie J.-B. Baillière et Fils, 1876).

[45] Darwin, C. *The Expression of Emotion in Man and Animals* (John Murray, 1872).

[46] Morecraft RJ, Cipolloni PB, Stilwell-Morecraft KS, Gedney MT, Pandya DN (2004). Cytoarchitecture and cortical connections of the posterior cingulate and adjacent somatosensory fields in the rhesus monkey. J. Comp. Neurol..

[47] Ross ED, Prodan CI, Monnot M (2007). Human facial expressions are organized functionally across the upper-lower facial axis. The Neuroscientist.

[48] Shepherd SV, Freiwald WA (2018). Functional networks for social communication in the macaque monkey. Neuron.

[49] Gothard K (2014). The amygdalo-motor pathways and the control of facial expressions. Front. Neurosci..

[50] Tiruvadi, V. et al. Mitigating mismatch compression in differential local field potentials. *IEEE Trans. Neural Syst. Rehabil*. *Eng.*10.1109/TNSRE.2022.3217469 (2022). 10.1109/TNSRE.2022.3217469 PMC10784110 36288215

[51] Stanslaski S (2012). Design and validation of a fully implantable, chronic, closed-loop neuromodulation device with concurrent sensing and stimulation. IEEE Trans. Neural Syst. Rehabil. Eng..

[52] Rokem, A., Trumpis, M. & Perez, F. Nitime: time-series analysis for neuroimaging data. In *Proceedings of the 8th Python in Science Conference* (eds Varoquaux, G. et al.) 68–75 (2009).

[53] Dupré la Tour T (2017). Non-linear auto-regressive models for cross-frequency coupling in neural time series. PLoS Comput. Biol..

[54] Tort ABL, Komorowski R, Eichenbaum H, Kopell N (2010). Measuring phase-amplitude coupling between neuronal oscillations of different frequencies. J. Neurophysiol..

[55] Paszke, A. et al. in *Advances in Neural Information Processing Systems* Vol. 32 (eds Wallach, H. M. et al.) 7994–8006 (Curran Associates, 2019).

[56] Kingma, D. P. & Welling, M. Auto-encoding variational Bayes. Preprint at *ArXiv*, 10.48550/arXiv.1312.6114 (2014).

[57] Jenkinson M, Beckmann CF, Behrens TEJ, Woolrich MW, Smith SM (2012). FSL. NeuroImage.

[58] Dunlop BW (2017). Functional connectivity of the subcallosal cingulate cortex and differential outcomes to treatment with cognitive-behavioral therapy or antidepressant medication for major depressive disorder. Am. J. Psychiatry.

[59] Smith SM (2006). Tract-based spatial statistics: Voxelwise analysis of multi-subject diffusion data. NeuroImage.

[60] Noecker AM (2018). StimVision software: examples and applications in subcallosal cingulate deep brain stimulation for depression. Neuromodulation J. Int. Neuromodulation Soc..

[61] Warrington S (2020). XTRACT - standardised protocols for automated tractography in the human and macaque brain. NeuroImage.

[62] Baltrušaitis, T., Robinson, P. & Morency, L.-P. OpenFace: an open source facial behavior analysis toolkit. In *2016 IEEE Winter Conference on Applications of Computer Vision (WACV)*, 51–60 10.1109/WACV.2016.7477553 (IEEE, 2016).

[63] Chawla NV, Bowyer KW, Hall LO, Kegelmeyer WP (2002). SMOTE: synthetic minority over-sampling technique. J. Artif. Intell. Res..

[64] Pedregosa F (2011). Scikit-learn: machine learning in Python. J. Mach. Learn. Res..

[65] Cheong, J. H., Xie, T., Byrne, S. & Chang, L. J. Py-Feat: Python facial expression analysis toolbox. Preprint at *ArXiv*, 10.48550/arXiv.2104.03509 (2021) 10.1007/s42761-023-00191-4 PMC10751270 38156250

[66] Kuznetsova A, Brockhoff PB, Christensen RH (2017). B. lmerTest package: tests in linear mixed effects models. J. Stat. Softw..

[67] Molnar, C. *Interpretable Machine Learning* (Lulu.com, 2020).

