# Supplementary material for: Cingulate dynamics track depression recovery with deep brain stimulation
Source: Nature. 2023 Sep 20;622(7981):130–8. doi: 10.1038/s41586-023-06541-3 (PMC10550829; doi:10.1038/s41586-023-06541-3)
Supplement: Supplementary file 1 — Reporting Summary [file 41586_2023_6541_MOESM1_ESM.pdf]

nature portfolio

Corresponding author(s): Christopher Rozell, Helen Mayberg

Last updated by author(s): 6/2/2023

Reporting Summary

Nature Portfolio wishes to improve the reproducibility of the work that we publish. This form provides structure for consistency and transparency in reporting. For further information on Nature Portfolio policies, see our Editorial Policies and the Editorial Policy Checklist.

Statistics

For all statistical analyses, confirm that the following items are present in the figure legend, table legend, main text, or Methods section.

n/a	Confirmed	
☐	☒	The exact sample size (*n*) for each experimental group/condition, given as a discrete number and unit of measurement
☐	☒	A statement on whether measurements were taken from distinct samples or whether the same sample was measured repeatedly
☐	☒	The statistical test(s) used AND whether they are one- or two-sided<br>*Only common tests should be described solely by name; describe more complex techniques in the Methods section.*
☐	☒	A description of all covariates tested
☐	☒	A description of any assumptions or corrections, such as tests of normality and adjustment for multiple comparisons
☐	☒	A full description of the statistical parameters including central tendency (e.g. means) or other basic estimates (e.g. regression coefficient) AND variation (e.g. standard deviation) or associated estimates of uncertainty (e.g. confidence intervals)
☐	☒	For null hypothesis testing, the test statistic (e.g. F, t, r) with confidence intervals, effect sizes, degrees of freedom and P value noted<br>*Give P values as exact values whenever suitable.*
☒	☐	For Bayesian analysis, information on the choice of priors and Markov chain Monte Carlo settings
☒	☐	For hierarchical and complex designs, identification of the appropriate level for tests and full reporting of outcomes
☐	☒	Estimates of effect sizes (e.g. Cohen's *d*, Pearson's *r*), indicating how they were calculated

Our web collection on statistics for biologists contains articles on many of the points above.

Software and code

Policy information about availability of computer code

Data collection	LFPs were acquired using Medtronic's proprietary Activa PC+S 8180 Sensing Software
Data analysis	All LFP analyses were performed using custom-written scripts in Python (v3.6) and Matlab (R2018b)<br>Spectral power and magnitude-squared coherence were estimated using the python library Nitime's (Rokem, Trumpis, and Perez 2009) (v0.9) multi-taper fast Fourier transform approach<br>Phase-amplitude coupling (PAC) was estimated using the PACtools python library (v0.3.1)<br>Neural network models were used to classify LFP features using PyTorch (v1.11.0)<br>Facial landmarks were extracted and secondary features were computed using Openface facial behavior analysis toolkit V2.0<br>A logistic regression classifier for facial expression analysis was implemented in the python sklearn (v1.1.1)<br>Imaging data was preprocessed using FMRIB Software Library v6.0, Analysis of Functional NeuroImages (AFNI) v23.1.06

For manuscripts utilizing custom algorithms or software that are central to the research but not yet described in published literature, software must be made available to editors and reviewers. We strongly encourage code deposition in a community repository (e.g. GitHub). See the Nature Portfolio guidelines for submitting code & software for further information.

Data

Policy information about availability of data

All manuscripts must include a data availability statement. This statement should provide the following information, where applicable:

- Accession codes, unique identifiers, or web links for publicly available datasets
- A description of any restrictions on data availability
- For clinical datasets or third party data, please ensure that the statement adheres to our policy

The data that support the findings of this study are publicly available via the Data Archive for The Brain Initiative (DABI) at https://dabi.loni.usc.edu/dsi/1UH3NS103550/UXUF7822Z3JL.

Field-specific reporting

Please select the one below that is the best fit for your research. If you are not sure, read the appropriate sections before making your selection.

☒ Life sciences ☐ Behavioural & social sciences ☐ Ecological, evolutionary & environmental sciences

For a reference copy of the document with all sections, see nature.com/documents/nr-reporting-summary-flat.pdf

Life sciences study design

All studies must disclose on these points even when the disclosure is negative.

Sample size	We used all the subjects (N = 6) for whom uncorrupted LFP data was available. The classifier performance was verified with leave-one-participant out cross validation. So conclusions were made for each participant individually. This open label study was powered based on past outcome experience (Riva Posse 2018) and availability of prototype devices (n=10) from the device manufacturer. Since this is a first-of-its kind study investigating LFP collected longitudinally during SCC DBS, it was not possible to power the study based on LFP outcomes
Data exclusions	Two participants were excluded from analysis as they had LFP data distorted by an amplifier clipping artifact (one participant) or heartbeat artifacts (one participant). These artifacts were identified by visual inspection of the LFP and spectra
Replication	Identification of electrophysiological changes was verified with leave-one-participant out cross validation thereby replicating finding in each individual participant. In addition, one participant whose data was not used in training or cross validation of the model supported the conclusions.
Randomization	Not applicable to the study. All participants received the same intervention.
Blinding	Not applicable to the study. This is an open-labeled study with the aim of investigating electrophysiological changes with no group allocation.

Reporting for specific materials, systems and methods

We require information from authors about some types of materials, experimental systems and methods used in many studies. Here, indicate whether each material, system or method listed is relevant to your study. If you are not sure if a list item applies to your research, read the appropriate section before selecting a response.

Materials & experimental systems

n/a	Involved in the study	
☒	☐	Antibodies
☒	☐	Eukaryotic cell lines
☒	☐	Palaeontology and archaeology
☒	☐	Animals and other organisms
☐	☒	Human research participants
☐	☒	Clinical data
☒	☐	Dual use research of concern

Methods

n/a	Involved in the study	
☒	☐	ChIP-seq
☒	☐	Flow cytometry
☐	☒	MRI-based neuroimaging

Human research participants

Policy information about studies involving human research participants

Population characteristics	18 Years to 70 Years, all sexes,<br>Current depressive episode of at least two years duration OR a history of more than 4 lifetime depressive episodes,<br>Failure to respond to a minimum of four different antidepressant treatments

Recruitment	Subject enrollment followed predefined inclusion and exclusion criteria (listed in Clinicaltrials.gov) that were similar to the ones used in other studies investigating invasive and non-invasive neuromodulation in treatment-resistant depression. Referrals could be made by psychiatrists in the community or in the Department of Psychiatry and Behavioral Sciences at Emory University School of Medicine. All subjects were initially examined by study psychiatrists, and an independent psychiatrist not involved in the study performed a separate evaluation to confirm selection. A consensus discussion was conducted as the final step before the invitation to participate in the study. As part of standard COI management, author HM did not participate in patient selection due to relevant intellectual property interests. In addition to the strict clinical criteria, other factors might have contributed to the selection of these subjects, such as proximity to the clinical and research site, availability for scheduling of frequent clinical visits, as well as transportation and family support. Study subjects were required to live in the greater Atlanta metropolitan area for a period of time due to the intensive clinical and experimental schedule.
Ethics oversight	Emory University Institutional Review Board; Emory University Department of Psychiatry and Behavioral Sciences Data and Safety Monitoring Board

Note that full information on the approval of the study protocol must also be provided in the manuscript.

Clinical data

Policy information about clinical studies

All manuscripts should comply with the ICMJE guidelines for publication of clinical research and a completed CONSORT checklist must be included with all submissions.

Clinical trial registration	Clincaltrials.gov - NCT01984710
Study protocol	Described in Molecular Psychiatry “A connectomic approach for subcallosal cingulate deep brain stimulation surgery: prospective targeting in treatment-resistant depression.<br>Riva-Posse P, Choi KS, Holtzheimer PE, Crowell AL, Garlow SJ, Rajendra JK, McIntyre CC, Gross RE, Mayberg HS.<br>Mol Psychiatry. 2018 Apr;23(4):843-849. doi: 10.1038/mp.2017.59. Epub 2017 Apr 11. PMID: 28397839”
Data collection	LFP and clinical assessments were collected during weekly sessions with clinicians in the study at Emory University, Atlanta GA
Outcomes	Activa PC+S LFP recordings - Continuous LFPs from SCC25 will be the primary dependent measure sampled throughout the study. Clinical outcome will be measured using the Hamilton Depression Rating Scale (HDRS) (17 item). Both the LFP and HDRS and Activa PC+S will be measured weekly for 4 weeks then weekly or biweekly for the next 3 months and monthly for 3 months every 3 months for 9 months then 6 month -12 months for 10 years. When the Activa PC+S battery is depleted which is anticipated to be after approximately 2-3 years there will be no more Activa PC+S outcome measures.

Magnetic resonance imaging

Experimental design

Design type	resting state fMRI
Design specifications	7.4 minutes
Behavioral performance measures	Resting-state fMRI, No behavioral performance measure

Acquisition

Imaging type(s)	High-resolution structural T1, diffusion-weighted, and resting-state fMRI images
Field strength	3 Tesla
Sequence & imaging parameters	magnetization-prepared rapid gradient-echo (MPRAGE) sequence with the following parameters: sagittal slice orientation; resolution=1.0mm×1.0 mm×1.0 mm; repetition time (TR)=2600ms; inversion time (TI)=900ms; echo time (TE)=3.02ms; flip angle=8°. DWI was acquired using single-shot spin-echo echo-planar imaging (EPI) sequence. Resting-state fMRI data was acquired using a Z-SAGA sequence with following parameters: 150 measurements; 30 axial slices; voxel resolution=3.4×3.4×4 mm3; matrix=64×64, TR/TE=2,950 ms/30 ms.
Area of acquisition	Whole brain

Diffusion MRI ☒ Used ☐ Not used

Parameters	DWI was acquired using single-shot spin-echo echo-planar imaging (EPI) sequence with the following parameters: 64 non-collinear directions with five non-diffusion weighted images (b0), b-value=1000sec/mm2; number of slices=64; field of view=256×256mm2; voxel size=2×2×2 mm3; TR=11300ms; TE=90ms.

Preprocessing

Preprocessing software	FMRIB Software Library v6.0, Analysis of Functional NeuroImages (AFNI) v23.1.06
Normalization	T1 image was skull stripped and normalized to MNI152 template using fsl_anat toolbox. Individual FA images were aligned to

Normalization

the standard FMRIB58 FA template using a nonlinear registration. The T1 image and fMRI data were co-registered using align_epi_anat.py in AFNI toolbox and normalized to MNI152 template using normalization parameters from T1 image.

Normalization template

T1 and fMRI images: MNI152 template, FA image: FMRIB58 FA

Noise and artifact removal

DWI data underwent distortion and motion collection using the Eddy toolbox and a local tensor fitting to calculate the FA map. Resting-state fMRI data were despiked and corrected for motion and slice-time acquisition and the remaining effects of the noise signal, including residual head motion inferences, signal from the CSF and local white matter, were removed. Subsequently, fMRI data were band-pass filtered and spatially smoothed up to 8 mm full-width at half-maximum using 3dBlurToFWHM in AFNI.

Volume censoring

3dDespike in AFNI

Statistical modeling & inference

Model type and settings

Within the specific tracks of FA skeleton, Spearman's rank correlation between FA and the inferred transition times was performed to evaluate whether WM microstructure at baseline could predict the inferred transitions in states. The same analyses were done in a whole-brain voxel-wise for the resting-state functional connectivity using the bilateral SCC seeds. The threshold was set at uncorrected p<0.05.

Effect(s) tested

N/A

Specify type of analysis: ☐ Whole brain ☒ ROI-based ☐ Both

Anatomical location(s)

White matter tracts passing through VTA were extracted in each subject using the Xtract toolbox in FSL (Warrington et al. 2020) and then averaged to generate a white matter tract mask that represents common activation pathways of all five subjects. Three white matter masks, including forceps minor (FM), cingulum bundle (CB), and uncinate fasciculus (UF), were included for the statistical analysis

Statistic type for inference
(See Eklund et al. 2016)

voxel-wise uncorrected p < 0.05

Correction

N/A

Models & analysis

n/a	Involved in the study
☐	☒ Functional and/or effective connectivity
☒	☐ Graph analysis
☒	☐ Multivariate modeling or predictive analysis

Functional and/or effective connectivity

The mean time course of the bilateral SCC seed was correlated voxel-wise with the rest of the brain using Pearson's correlation coefficient. The correlation coefficient maps were converted to z-value using Fisher's r-to-z transformation.
